# Supplementary material for: Beyond structural bioinformatics for genomics with dynamics characterization of an expanded KRAS mutational landscape
Source: Comput Struct Biotechnol J. 2023 Oct 5;21:4790–803. doi: 10.1016/j.csbj.2023.10.003 (PMC10570560; doi:10.1016/j.csbj.2023.10.003)
Supplement: Supplementary file 1 — Supplementary material [file mmc1.docx]

# SUPPLEMENTAL INFORMATION

**Table S1: The number of experimentally solved KRAS structures, per mutational and ligand status.**

| Mutation | Nucleotide | Fully Resolved (1-169) | In complex | Not Fully Resolved | In complex |
| --- | --- | --- | --- | --- | --- |
| WT | GTP | 17 | 6 | 10 | 9 |
|  | GDP | 22 | 6 | 12 | 3 |
|  | apo | - | - | 1 | 1 |
| G12C | GTP | - | - | 1 | - |
|  | GDP | 28 | - | 15 | - |
|  | apo | - | - | 5 | 5 |
| G12D | GTP | 12 | 1 | 3 | - |
|  | GDP | 2 | - | 6 | 2 |
|  | apo | - | - | - | - |
| Q61H | GTP | 7 | - | 1 | - |
|  | GDP | - | - | - | - |
|  | apo | - | - | - | - |
| G12V | GTP | 1 | 1 | 4 | - |
|  | GDP | 5 | - | 2 | - |
|  | apo | - | - | - | - |
| G13D | GTP | 3 | 3 | 1 | - |
|  | GDP | 2 | - | - | - |
|  | apo | - | - | - | - |
| Q61R | GTP | 2 | 2 | - | - |
|  | GDP | - | - | - | - |
|  | apo | - | - | - | - |
| P34R | GTP | 2 | - | 1 | - |
|  | GDP | - | - | 1 | - |
|  | apo | - | - | - | - |
| G12A | GTP | - | - | 3 | - |
|  | GDP | 1 | - | 1 | - |
|  | apo | - | - | - | - |
| G12R | GTP | - | - | 1 | - |
|  | GDP | 1 | - | - | - |
|  | apo | - | - | - | - |
| V14I | GTP | - | - | - | - |
|  | GDP | 1 | - | 1 | - |
|  | apo | - | - | - | - |
| A146T | GTP | - | - | - | - |
|  | GDP | 1 | - | - | - |
|  | apo | - | - | - | - |
| A49G | GTP | 1 | - | - | - |
|  | GDP | - | - | - | - |
|  | apo | - | - | - | - |
| A59G | GTP | - | - | - | - |
|  | GDP | 1 | - | - | - |
|  | apo | - | - | - | - |
| D33E | GTP | - | - | - | - |
|  | GDP | 1 | - | - | - |
|  | apo | - | - | - | - |
| Q61L | GTP | - | - | 1 | - |
|  | GDP | - | - | 1 | - |
|  | apo | - | - | - | - |
| Q61A | GTP | - | - | - | - |
|  | GDP | 1 | - | - | - |
|  | apo | - | - | - | - |
| M72C | GTP | - | - | - | - |
|  | GDP | 1 | - | - | - |
|  | apo | - | - | - | - |

**Table S2: Complete list of scores on a per variants basis (Additional File)**


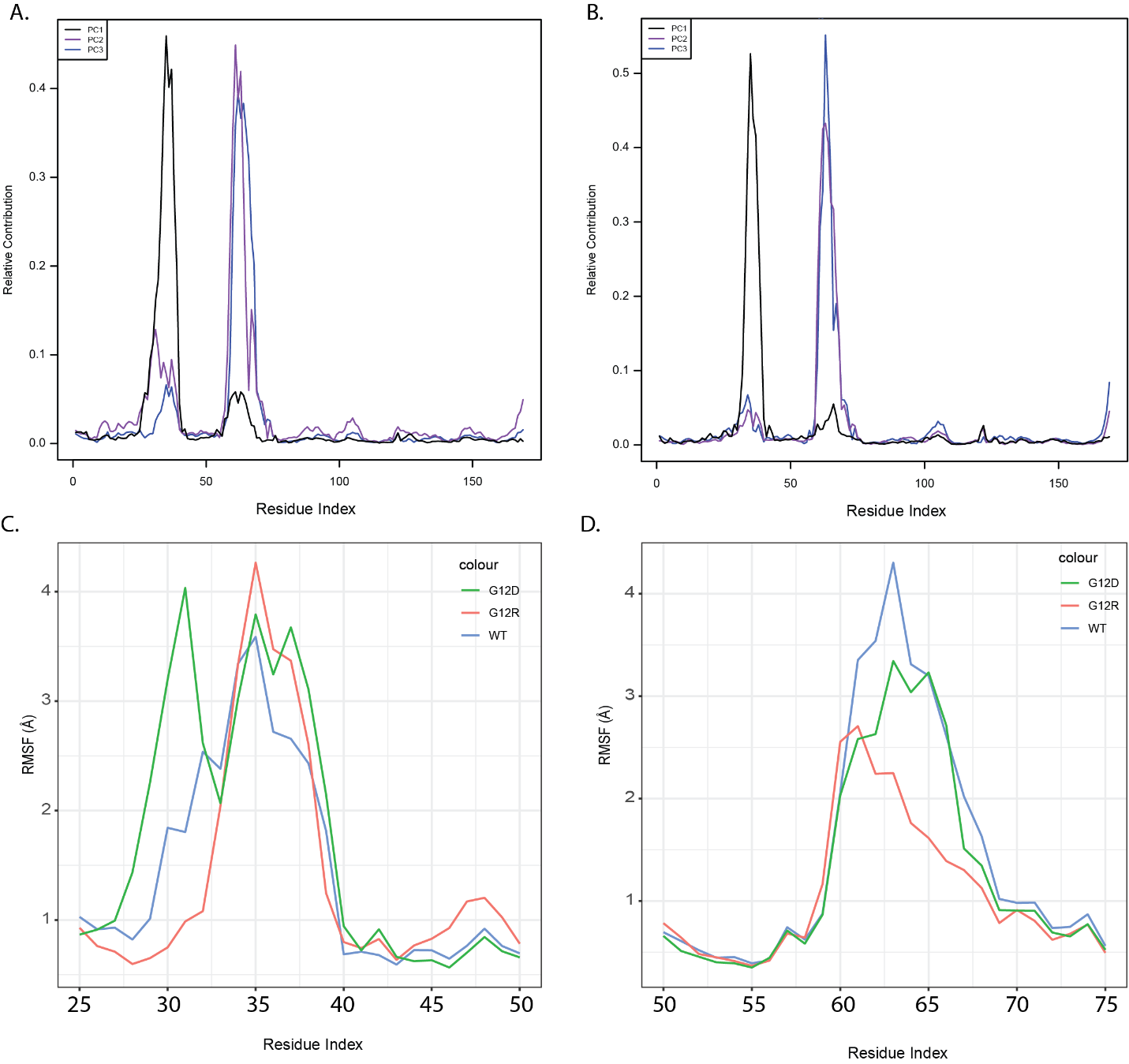


**Figure S1: PCA relative contributions** for **A**) GTP and **B**) GDP simulated coordinate space. GDP simulation RMSF of **C**) Switch 1 and **D**) Switch 2 for WT, G12R, and G12D.


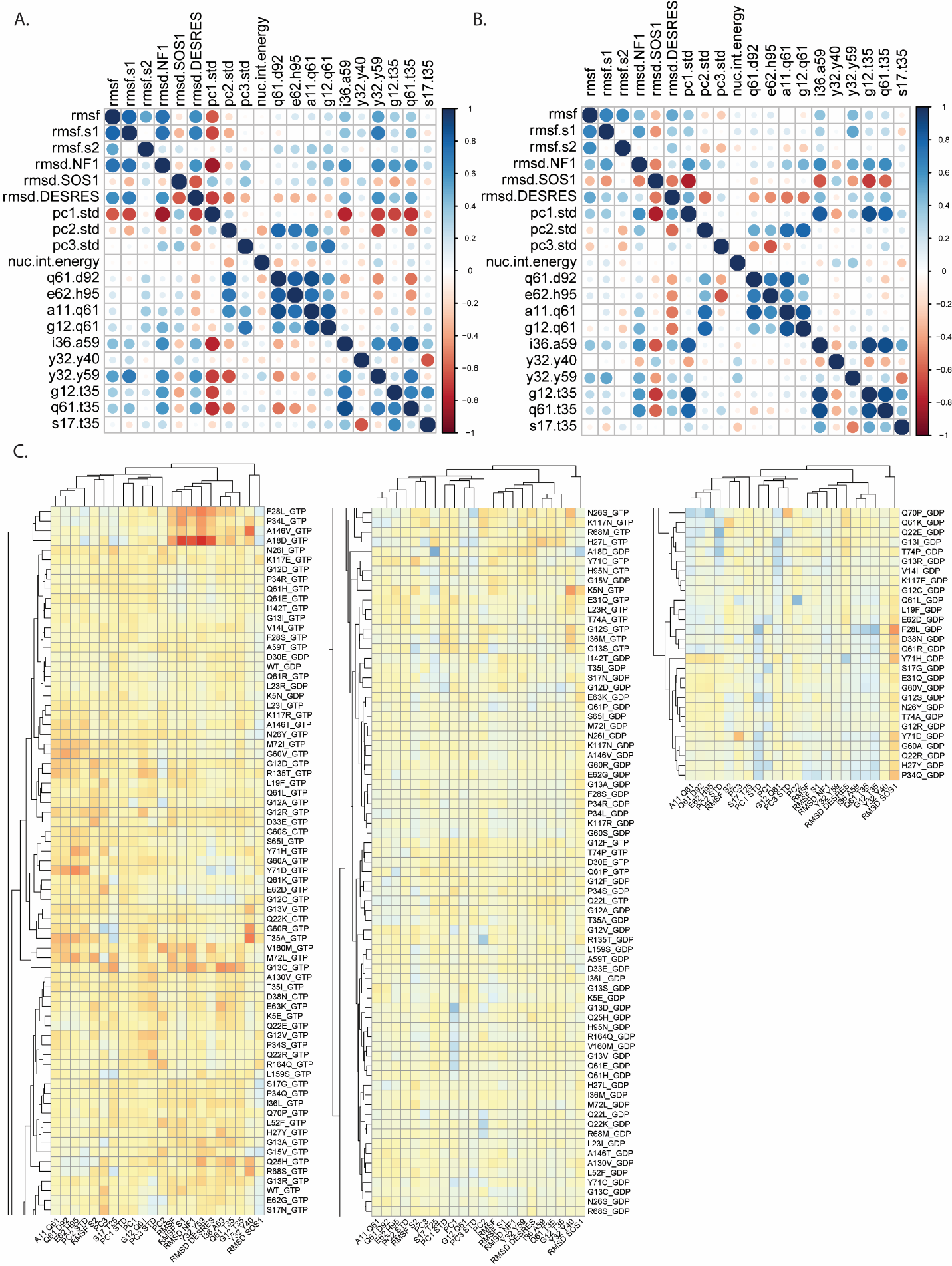


**Figure S2: Summary of interconnectedness between MD metrics.** Correlation matrix between all MD metrics for **A**) GDP and **B**) GTP simulations. **C**) Heatmap of all score values on a per variant basis. Red values indicate higher score values, while blue values indicate of lower score values. Scores are clustered via k-means on the x-axis and variants are clustered via k-means on the y-axis.


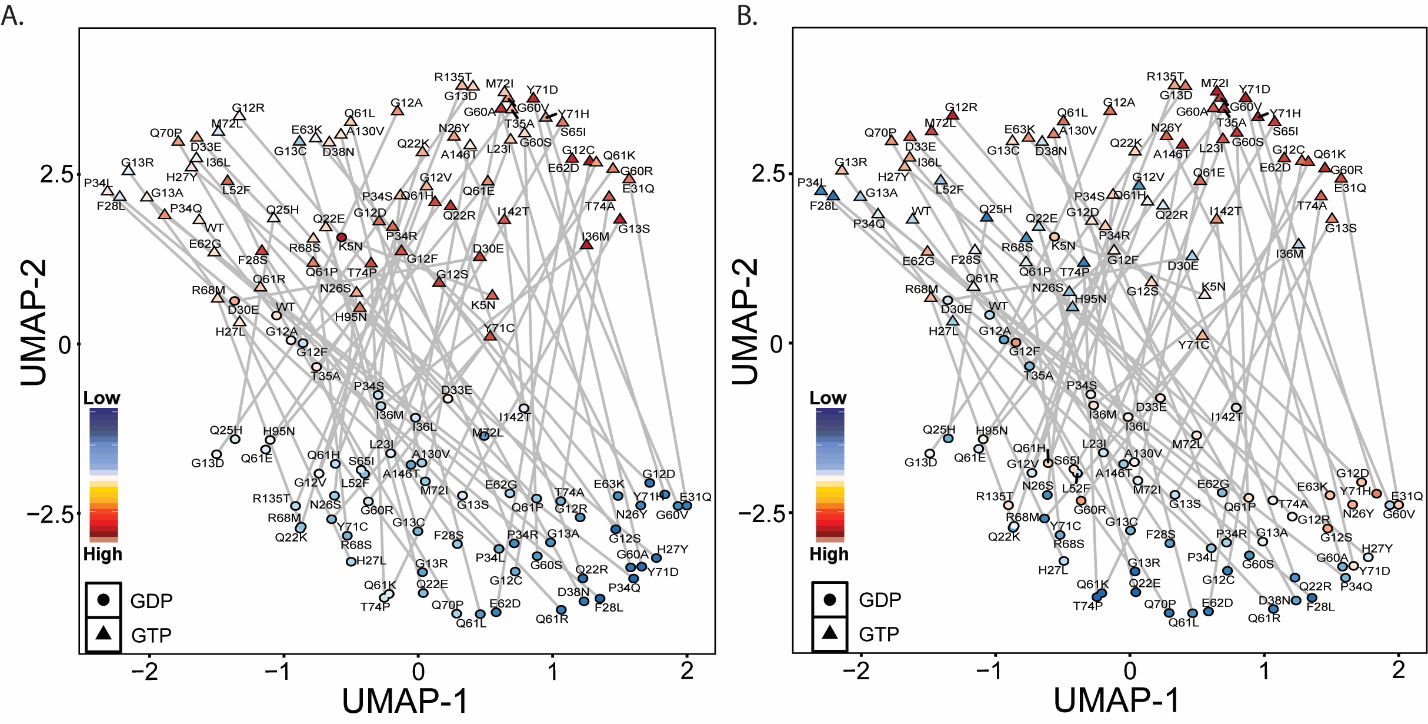


**Figure S3: Observing PC Standard Scores over UMAP exemplifies the relative importance of features.**

**A**) UMAP dimensionality reduction calculated using all MD metrics discussed in this study. Each variant is colored according to the PC1 standard score and using the same format as Figure 4A. and **B**) according to the PC2 standard score.
